# What are the characteristics that lead physicians to perceive an ICU stay as non-beneficial for the patient?

**DOI:** 10.1371/journal.pone.0222039

**Published:** 2019-09-06

**Authors:** Jean-Pierre Quenot, Audrey Large, Nicolas Meunier-Beillard, Paul-Simon Pugliesi, Pamina Rollet, Amaury Toitot, Pascal Andreu, Hervé Devilliers, Antoine Marchalot, Fiona Ecarnot, Auguste Dargent, Jean-Philippe Rigaud, on behalf of the INSTINCT study group

**Affiliations:** 1 Department of Intensive Care, François Mitterrand, University Hospital, Dijon, France; 2 Lipness Team, INSERM Research Centre LNC-UMR1231 and LabEx LipSTIC, University of Burgundy, Dijon, France; 3 INSERM CIC 1432, Clinical Epidemiology, University of Burgundy, Dijon, France; 4 DRCI, USMR, Francois Mitterrand University Hospital, Dijon, France; 5 Department of Intensive Care, William Morey Hospital, Chalon sur Saône, France; 6 Department of Intensive Care, Nord Franche-Comté Hospital, Trevenans, France; 7 Department of Internal Medicine, François Mitterrand University Hospital, Dijon, France; 8 Department of Intensive Care, Dieppe General Hospital, Dieppe, France; 9 EA3920, Department of Cardiology, University Hospital Besancon, France; 10 Espace de Réflexion Ethique de Normandie, University Hospital Caen, France; University of Auckland, NEW ZEALAND

## Abstract

**Purpose:**

We sought to describe the characteristics that lead physicians to perceive a stay in the intensive care unit (ICU) as being non-beneficial for the patient.

**Materials and methods:**

In the first step, we used a multidisciplinary focus group to define the characteristics that lead physicians to consider a stay in the ICU as non-beneficial for the patient. In the second step, we assessed the proportion of admissions that would be perceived by the ICU physicians as non-beneficial for the patient according to our focus group’s definition, in a large population of ICU admissions in 4 French ICUs over a period of 4 months.

**Results:**

Among 1075 patients admitted to participating ICUs during the study period, 155 stays were considered non-beneficial for the patient, yielding a frequency of 14.4% [95% confidence interval (CI) 8.9, 19.9]. Average age of these patients was 72 ±12.8 years. Mortality was 43.2% in-ICU [95%CI 35.4, 51.0], 55% [95%CI 47.2, 62.8] in-hospital. The criteria retained by the focus group to define a non-beneficial ICU stay were: patient refusal of ICU care (23.2% [95%CI 16.5, 29.8]), and referring physician’s desire not to have the patient admitted (11.6% [95%CI 6.6, 16.6]). The characteristics that led physicians to perceive the stay as non-beneficial were: patient’s age (36.8% [95%CI 29.2, 44.4]), unlikelihood of recovering autonomy (61.9% [95%CI 54.3, 69.6]), prior poor quality of life (60% [95%CI 52.3, 67.7]), terminal status of chronic disease (56.1% [95%CI 48.3, 63.9]), and all therapeutic options have been exhausted (35.5% [95%CI 27.9, 43.0]). Factors that explained admission to the ICU of patients whose stay was subsequently judged to be non-beneficial included: lack of knowledge of patient’s wishes (52% [95%CI 44.1, 59.9]); decisional incapacity (sedation) (69.7% [95%CI 62.5, 76.9]); inability to contact family (34% [95%CI 26.5, 41.5]); pressure to admit (from family or other physicians) (50.3% [95%CI 42.4, 58.2]).

**Conclusions:**

Non-beneficial ICU stays are frequent. ICU admissions need to be anticipated, so that patients who would yield greater benefit from other care pathways can be correctly oriented in a timely manner.

## Introduction

In the daily routine practice of Intensive Care Unit (ICU) physicians, established criteria usually make it possible for admission decisions to be made unequivocally [1, 2]. However, the recommendations of the Society of Critical Care Medicine (SCCM) for ICU triage, admission, and discharge [1] suggest that some overtriage is acceptable, i.e. the understanding is that it will inevitably transpire that some patients who were admitted to the ICU could have been adequately treated via another care pathway without requiring ICU care. This type of situation may arise in particular when the ICU admission occurs in the context of an acute, unanticipated episode, where the patients’ wishes may be unknown, and/or the family are unavailable [3].

Once a patient has been admitted to the ICU, and intensive care is being delivered, physicians may rapidly perceive that the stay in the ICU is non-beneficial for that patient, the principle of beneficence notwithstanding. The reasons that lead physicians to perceive an ICU stay as being non-beneficial are unclear, and the perception that the stay is non-beneficial does not necessarily mean that there is a failure to comply with guidelines for appropriate management. The SCCM guidelines suggest “avoiding the current quantitative definitions of non-beneficial treatment because of the lack of consensus on a single definition”, and no grade of evidence is given for this recommendation in the absence of extensive literature. Clearly, there is a compelling need for a qualitative evaluation of the patient-, and/or situation-related characteristics that lead clinicians to consider an ICU stay as being non-beneficial for a given patient.

Against this background, the objective of the current study was threefold: (1) to define the characteristics that lead physicians to perceive a stay in the intensive care unit (ICU) as being non-beneficial for the patient using a focus group method; (2) to apply this definition in a large sample of ICU admissions, in order to assess the proportion of admissions that would be perceived by the ICU physicians as non-beneficial according to the focus group definition; and finally (3) to investigate the circumstances that led to such patients being admitted to the ICU.

## Methods

This is an exploratory, observational, prospective, multicentre study performed in two stages.

In a first stage, we convened a multidisciplinary focus group to establish criteria defining non-beneficial admissions. In the second stage, we investigated all ICU admissions to identify non-beneficial admissions over a period of 4 months We considered consecutively admitted patients aged 18 years or older, with a theoretical indication for intensive care (need for life-support therapy, i.e. mechanical ventilation, renal replacement therapy, vasopressors) in 4 hospitals in France (1 university teaching hospital and 3 non-academic general hospitals). The study was performed from 1 March to 1 July 2018.

### Ethics approval and consent to participate

The institutional review board (Comité de Protection des Personnes Est I, Dijon) approved the protocol, and considered it to constitute routine clinical practice. The need for informed consent was waived, but all patients or their relatives were given clear information about the study, and their non-opposition was obtained. Collection of nominative data was approved by the national authority for the protection of privacy and personal data.

### Focus group

A focus group was constituted, comprising 10 physicians working in the ICU of academic and/or non-academic hospitals, and a sociologist who cares for critically ill patients. The physicians were senior ICU physicians with experience in critical care and in the field of medical ethics. The group discussed the criteria that would lead them to consider ICU admission to be non-beneficial for a specific patient. This type of focus group methodology has previously been used in similar contexts [4, 5]. Based on a review of the literature, one clinician (JPQ) and one health sociologist (NMB) developed an interview guide with open questions on the theme of “non-beneficial ICU stays”. The same two authors led the discussion, using open-ended questions. The focus group members were asked to describe patients for whom they had endorsed ICU admission that they judged to be non-beneficial. They were asked what made them view the admission to the ICU as non-beneficial, how a case perceived to be non-beneficial differed from other cases, and exactly when, during the course of the admission, they realized that the admission was non-beneficial. Participants were further asked to classify the reasons for non-beneficial admission to the ICU. Audiotapes of the discussion were transcribed and analyzed independently by two researchers (JPQ, NMB) using N Vivo software (version 10) for data management. The transcripts were returned to participants from focus group to comment on the accuracy of the data. Criteria for non-beneficial admission were defined after approval of the transcripts by all the focus group members.

### Survey instrument

On the basis of these discussions, the criteria defining non-beneficial admission were compiled into a questionnaire to be applied to each ICU admission in the second stage of the study to identify patients whom the physicians perceived as receiving non-beneficial ICU admission. For each ICU patient under the physician’s care, the questionnaire was completed during a collegial discussion with the healthcare team, to decide whether the patient was receiving non-beneficial treatment. For admissions judged to be non-beneficial for the patient, the physician was asked to select the reason(s) why it was non-beneficial, from among the list of criteria defined by the Focus Group, namely: the patient had declined ICU care (existence of advance directives or other wishes formulated orally to the family and/or surrogate); the referring physician(s) did not want the patient to be admitted to the ICU in case of an acute event; the medical team deemed the admission of the patient to be disproportionate or unreasonable (advanced age, severely limited autonomy, poor quality of life, terminal stage of chronic disease, therapeutic impasse). We also sought to identify the reasons that nonetheless motivated the admission to ICU, such as lack of knowledge about the patient’s wishes, decisionally incapacitated patient (e.g. brain damage, sedation), inability to contact any next of kin or surrogate, inability to contact the referring physician to obtain medical information about the patient, pressure from the family and/or physician in charge and/or referring physician, despite very poor prognosis, and the fear of legal action. Physicians also could write any other reason. The questionnaire was piloted for one month to test ease of administration, wording, and content.

### Administration of the questionnaire

For each patient admitted to the ICU, from 1 March through 1 July 2018, the ICU physician completed the study questionnaire. Patients were eligible if they were admitted to the ICU and required life-support therapy during their stay for failure of at least one major organ. Patients not requiring any life-support therapy were not considered. Patients who were refused ICU admission were also not considered in this study.

During the staff meetings, the caregivers (physicians, nurses) were invited to participate (if they were available) to discuss each individual case, and specifically the patients under their care. The junior physician, under the supervision of the senior physician, presented the patient’s case after having recorded the maximum of medical information pertaining to the patient (level of autonomy, quality of life, comorbidities, presence of chronic disease, therapeutic outlook, prognosis, patient’s wishes regarding resuscitation, in particular advance directives). The questionnaire was completed within 48 to 72 hours of admission, making it possible to include patients admitted at the weekend or on holidays. The patient’s family were also met as soon as possible after admission to obtain information about the patient’s life trajectory. The patient’s referring physician(s) was (were) also contacted as soon as possible after admission to obtain the most accurate information possible about the patient’s general state of health, any ongoing disease, and therapeutic possibilities. The admission was considered non-beneficial if the physician(s) considered that the patient presented any one or more of the criteria defining non-beneficial admission. Patients could have more than one criteria, but the presence of one criterion was sufficient to classify the admission as non-beneficial.

### Data sources

The following data were recorded for each patient: socio-demographic characteristics, main reason for admission in ICU, co-morbidities evaluated by the Charlson index [6], the Knaus Chronic Health Status score consisting of: Class A: normal health status, Class B: moderate activity limitation, Class C: severity activity limitation due to chronic disease, and Class D: bedridden patient [7]; severity of disease calculated using the Simplified Acute Physiology Score (SAPS) II [8]; life-support therapy in ICU, length of ICU and hospital stay, decision to withhold or withdraw ICU care; and in-ICU and in-hospital death. Dedicated clinical research assistants collected all data using a standardized electronic case report form. Automatic checks were generated for missing or incoherent data. Data was independently managed by the Centre for Clinical and Epidemiological Investigation (Centre d’Investigation Clinique et Epidémiologie Clinique, CIC 1432). Data were taken from the patients’ medical files and thus, there were no missing data.

### Statistical analysis

Qualitative variables are expressed as numbers (percentages) and quantitative data as medians (ranges) with interquartile range [IQR]. All analyses were performed using SAS version 9.1 (SAS Institute Inc, Cary, NC, USA).

## Results

**Fig 1:**
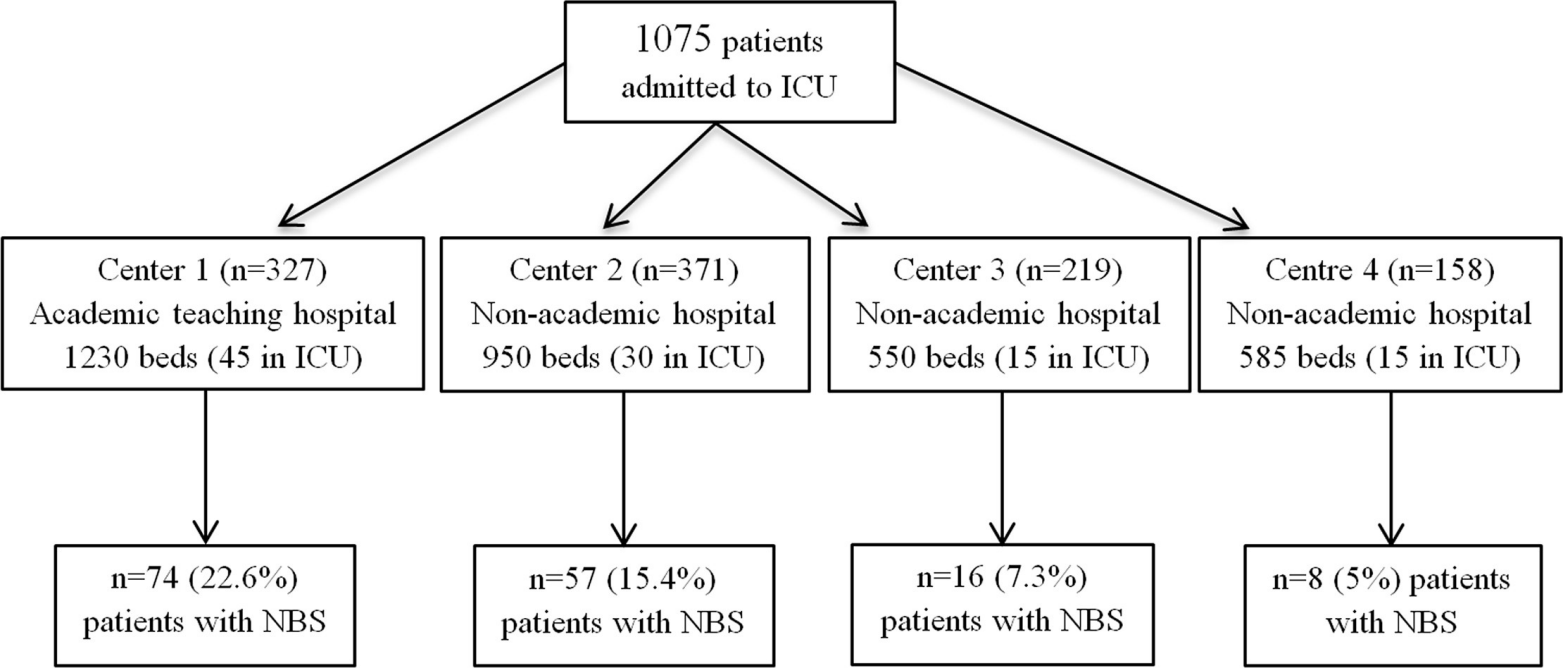
Flow chart of the participating centres and study population. NBS, non-beneficial stay in the intensive care unit.

Among 1075 admissions to the participating ICUs during the study period, 155 were considered to be non-beneficial for the patient, yielding a frequency of 14.4% of non-beneficial admissions [95% confidence interval (CI) 8.9, 19.9]. The distribution of the study population across the four centres is detailed in the study flowchart (Fig 1).

The main characteristics of the 155 patients with non-beneficial admission are given in Table 1. Average age was 72 ±12.8 years. Average Charlson comorbidity index was 3±1.9, and SAPS II 57±20. In total, 111 patients (71.6%) were severely limited in the activities of daily living (Knaus score C/D). Around two-thirds of patients included had life-support therapy, while for three-quarters of the population, a decision to limit or withdraw treatment was made in the ICU. Mortality in the ICU and in-hospital was respectively 43.2% [95%CI 35.4, 51.0], and 55% [95%CI 47.2, 62.8].

**Table 1:** Characteristics of the patients considered as “non-beneficial” admissions.

Variable	All (N = 155)
Age, years (mean±SD)	72±12.8
Male sex	98 (63.2%)
Main reason for ICU admission, n (% [95%CI])	
Respiratory	80 (51.6% [43.7, 59.5])
Sepsis	34 (21.9% [15.4, 28.4])
Cardiac	21 (13.5% [8.1, 18.9])
Renal	5 (3.2% [0.4, 5.9])
Neurological	4 (2.6% [0.1, 5.1])
Other reasons	11 (7% [2.9, 11.0])
Charlson comorbidity score	3±1.9
Knaus score, n (% [95%CI])	
A (normal health)	6 (3.9% [0.8, 6.9])
B (moderate activity limitation)	37 (23.9% [17.2, 30.6])
C (severe activity limitation)	90 (58.1% [50.3, 65.9])
D (bedridden patient)	21 (13.5% [8.1, 18.9])
SAPS II score	57±20
Life support use in ICU, n (% [95%CI])	
Mechanical ventilation	94 (60.6% [52.9, 68.3])
Vasopressors / inotropes	96 (61.9% [54.2, 69.5])
Renal replacement therapy	13 (8.4% [4.0, 12.8])
Median [IQR] length of ICU stay	4 [2, 8]
Median [IQR] length of hospital stay	12 [5, 21]
Decision to withdraw/withhold care in ICU, n (% [95%CI])	115 (74.2% [67.3, 81.1])
In-ICU Death	67 (43.2% [35.4, 51.0])
In-hospital Death	85 (55% [47.2, 62.8])

SD, Standard deviation; 95%CI, 95% confidence interval; ICU, intensive care unit; SAPS II, Simplified Acute Physiology Score II; IQR, interquartile range.

### Reasons qualifying admission as non-beneficial

Among the criteria defining ICU admission as non-beneficial, we noted patient refusal of ICU care (23.2% [95%CI 16.5, 29.8]), and the desire expressed by the referring physician(s) not to have the patient admitted to the ICU (11.6% [95%CI 6.6, 16.6]). In the vast majority of cases (98.1%), the medical team judged the patient’s admission to the ICU as non-beneficial, for the following reasons (more than once reason could be cited for each patient): age (36.8% [95%CI 29.2, 44.4]), unlikely to recover autonomy (61.9% [95%CI 54.3, 69.6]), prior poor quality of life (60% [95%CI 52.3, 67.7]), terminal status of chronic disease (56.1% [95%CI 48.3, 63.9]), and all therapeutic options have been exhausted (35.5% [95%CI 27.9, 43.0]).

### Factors associated with non-beneficial admission to the ICU

The mean time from hospital admission to ICU admission was 4.07 ±9.75 days. The following factors were cited as explanations for the non-beneficial admission to the ICU: lack of knowledge of the patient’s wishes (52% [95%CI 44.1, 59.9]); patient decisionally incapacitated, particularly because of sedation (69.7% [95%CI 62.5, 76.9]); inability to contact any family or next of kin (34% [95%CI 26.5, 41.5]); and pressure to admit (from the family or other physicians) (50.3% [95%CI 42.4, 58.2]).

## Discussion

To the best of our knowledge, this is one of the first multicentre studies to evaluate the frequency of, and reasons for an ICU stay being perceived as non-beneficial, using a focus group methodology. The main results show that ICU stays may frequently be perceived to be non-beneficial for the patient: 5 to 22.6% according to the centre. Limited autonomy, poor former quality of life, terminal illness, therapeutic impasse and the patient’s refusal to be admitted to the ICU were the arguments most commonly cited by the medical team to justify considering the admission as non-beneficial. External constraints (e.g. from other physicians and/or from the patient’s family), lack of knowledge of the patient’s wishes, and a lack of medical information were the main reasons cited to explain why those patients were admitted to the ICU. It should be noted that some of this information, such as the details of the patient’s wishes or the referring physician’s preferences, only became known after the patient’s admission, and was not available to the ICU physician at the time the admission was being decided.

There is a clear disparity between the frequency of non-beneficial admissions across the participating centres. The reasons for this could be explored in further studies, and probably stem from differences between the centres in terms of case mix, local policy, type and number of available continuous care or intensive care beds in the hospital, caregiver-to-ICU-bed ratio, oncological activity in the hospital or the existence of a cancer treatment centre in close proximity to the hospital.

In the literature, data are sparse regarding the frequency of non-beneficial ICU admissions. Azoulay et al showed in a French multicentre study that 26.1% of patients admitted to the ICU were in conditions that could lead the ICU stay to be considered as futile (e.g. vegetative state, brain death, metastatic disease without hope of remission, terminal respiratory or heart failure) [9]. Similarly, a study in the UK found that 17.2% of ICU physicians would have admitted to the ICU a patient whose probability of survival was estimated to be less than 1% [10]. In their prospective, single-centre study from Germany, Bangert et al reported that among the 50 patients for whom the ICU admission was considered to be futile by interdisciplinary consensus, 82% had expressed (either directly, or in writing through advanced directives, or through a family member) the desire not to be admitted to intensive care [11], whereas the corresponding percentage in our study was 23%. Cultural differences between countries, including legislative aspects, could at least partially explain these conflicting findings [12, 13].

In our study, the criteria used to qualify the ICU admission as non-beneficial were different to those used to describe non-beneficial treatments in the ICU. Indeed, in evaluating the beneficial or non-beneficial character of therapy in the ICU, the severity of disease and unfavourable course despite life-support are determining factors [4, 5, 9, 14]. Conversely, in our study, the focus group chose other criteria to define non-beneficial admission, in particular criteria that more closely resemble those used to refuse an admission to the ICU (apart from organisational considerations), based on the patient’s life trajectory, therapeutic project, and wishes regarding intensive care [1, 15, 16].

These criteria are coherent with the objectives for management that ICU physicians hold for the patients that are proposed for ICU care. These include, for example, avoiding non-beneficial admissions, limiting the use of life-support therapies whose mobilization might be considered disproportionate (invasive mechanical ventilation, dialysis etc), and sometimes, anticipating the question of an admission where the prognosis is clearly highly unfavourable. This supposes that the ICU physician be consulted, before acute organ failure occurs in a patient for whom the question of ICU admission might later arise [17–19]. We propose that the possibility of ICU admission should be included in the healthcare plan, in concertation with the patient, regardless of the stage of the disease the patient is suffering from. This approach could be noted in the patient’s medical file, albeit noting that the disease progresses over time, and so should the reflection about these complex situations. Indeed, in our study, there was an average of 4 days between hospital admission and ICU admission (with much longer durations in some patients). This time window represents an ideal opportunity to document the patient’s wishes for her/her further care in the medical file. This would help avoid situations where actions are taken that go against the patient’s wishes due to the patient’s wishes being unknown or the patient being unable to express him/herself. The ICU physician could have a role to play in this regard as an outside consultant, called upon by one or more referring physicians, and above all, by the patient, notably to guide the patient’s choices when expressing their wishes for end-of-life care, or formulating advance directives [20]. The “ideal” time for this process is clearly before an acute event occurs, and in any case, the physicians must strive to provide full, transparent and reliable information to the patient.

The issue of anticipation will without a doubt become a key challenge in the future. Indeed, it is of paramount importance to avoid non-beneficial admissions, for the sake of the patient and their family, since such admissions may lead to treatment or mobilization of therapeutic resources that are not appropriate. The potential psychological consequences also warrant investigation. In addition, the question of resource allocation must be addressed. Anticipating possible admissions by including intensive care in the patient’s healthcare plan, and defining (where necessary) the level of life-support to be engaged could actually contribute to more rational and equitable allocation of available resources [21–24]. Knowing the patient’s wishes, and those of the patient’s family, disposing of medical data in real time, and being aware of potential pressures were all aspects that this study found to be important for underpinning serious reflection about ICU admission, before the issue comes to a head.

In practical terms, to integrate this into routine practice without the administrative burden of the research environment, it would be useful to systematically record the patient’s wishes for future (including end-of-life) care as soon as they are admitted to the hospital. This could be documented in the medical file, for example. Particular attention should be paid to specific patient populations most likely to be concerned, such as polymorbid patients, those with progressive chronic diseases, and the oldest-old [19, 25]. A standardized, computerized form could be used, which could be made available in the patient’s file and in the hospital’s medical informatics records. Templates for advance directives forms have been proposed by several professional societies, including the French national health authority [26]. We also recommend that ICU physicians should be invited to participate in these discussions with the patient, to provide guidance and advice about what intensive care entails, when it may be needed, and how the patient might reasonably expect to benefit [20].

Various other approaches have been proposed to attempt to anticipate the question of ICU admission, such as advance care planning [27], ethics consultations or palliative care consultations [28–30]. However, it is evident that the ICU physician should remain the preferred point of contact for the decision on whether or not to admit a patient to the ICU, as well as for decisions on limiting or withdrawing life-support therapy [18, 31]. Indeed, the ICU physician, who is competent and knowledgeable about life-support options, is the best placed to evaluate the patient’s prognosis according to the presence or absence of organ failure [3, 32].

Our study presents some limitations that deserve to be underlined. Firstly, the number of centres was low, and the inclusion period was quite short, although we do not anticipate any seasonal variations in the type of patient admitted. The size of the population precludes any multivariate analysis of factors influencing non-beneficial admission. Secondly, our findings do not provide any explanation for the differences observed in the frequency of non-beneficial admissions, and also did not address the cost that they may represent, which would require a larger study. Thirdly, we did not include patients who had no organ failure, which likely led to an under-estimation of the true frequency of non-beneficial admissions. We also did not include patients who were refused admission to the ICU. Finally, we did not evaluate the potential psychological consequences for the patients, their families, or the healthcare professionals, of non-beneficial admission to intensive care.

## Conclusion

Non-beneficial ICU stays are frequent. There is a compelling need to discuss and anticipate the possibility of ICU admission-before an acute occurs, notably taking into account the patient’s wishes, therapeutic profile, and life trajectory.
